# Steroid Fingerprinting with Cryogenic Gas-Phase Infrared Spectroscopy

**DOI:** 10.1021/acsmeasuresciau.6c00043

**Published:** 2026-04-13

**Authors:** Caitlin Walton-Doyle, Gurpur Rakesh D. Prabhu, Niklas Geue, Gerard Meijer, Gert von Helden, Kevin Pagel

**Affiliations:** † Institute of Chemistry and Biochemistry, 9166 Freie Universität Berlin, Altensteinstraße 23a, 14195 Berlin, Germany; ‡ Department of Molecular Physics, 28259 Fritz-Haber-Institut der Max-Planck-Gesellschaft, Faradayweg 4-6, 14195 Berlin, Germany

**Keywords:** metabolites, ion mobility, gas-phase IR spectroscopy, steroids, density functional theory

## Abstract

Despite important applications in anti-doping testing and endocrinology, the differentiation and characterization of steroid structures remain an analytical challenge. A key difficulty is the presence of isomeric species with only subtle structural differences. Here, we analyze two sets of isomeric steroids: the corticosteroids, aldosterone and cortisone, as well as 11β-, 17α-, and 21-hydroxyprogesterone. When probed with ion mobility, the analytes exhibit little variation in collision cross section values on a Synapt G2-S, making their separation from mixtures challenging. Ion mobility also offers limited information about their gas-phase structures. Cryogenic gas-phase infrared spectroscopy provides an additional dimension for identification and distinguishes between the isomers, enabling definitive structural elucidation. In combination with density functional theory, we present experimental data on the preferred protonation of the C3 carbonyl group. Notably, for aldosterone, an unusual bicyclic structure was identified. Overall, this work showcases the value of gas-phase infrared spectroscopy for steroid differentiation and identification, potentially contributing to anti-doping measures and diagnostics for adrenal and congenital disorders.

## Graphical abstract

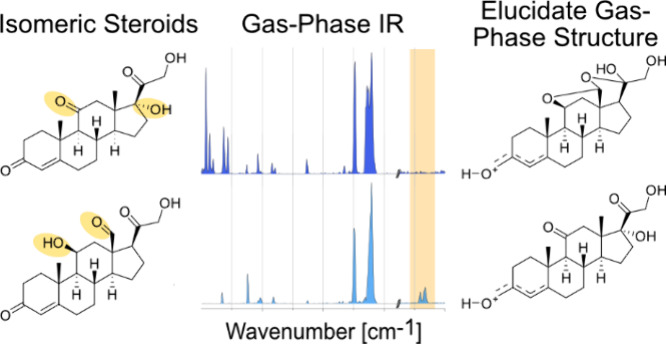

## Introduction

Steroids are structurally diverse biomolecules with profound physiological significance, functioning as essential hormones, mediators of immune responses,^1^ and, in some cases, used for illicit performance enhancement.^2^ In general, they are characterized by a core structure of 17 carbon atoms in four fused rings. Their function is highly dependent on their structure, with subtle changes in functional group position and stereochemistry leading to significant differences in their biological activity.^3^ This is, for example, evident in testosterone and epitestosterone, where only the former has strong androgenic effects,^4^ demonstrating the need for sensitive and specific methods that distinguish closely related steroids.

Due to their roles in biophysiological processes, steroids are commonly studied in metabolomics, which describes the global study of small molecule intermediates and products of metabolism in the body.^5,6^ A major bottleneck in metabolomics is the unambiguous identification of unknown structures due to limited databases and standards, variability across platforms, and structural isomerism.^7^ Properties such as protonation sites and tautomerization can further complicate the interpretation of spectra,^8^ and the complex biological matrices typically analyzed in omics studies can give large numbers of features.^9–14^ Metabolites, including steroids, are often analyzed by gas chromatography-mass spectrometry (GC-MS) and liquid chromatography-mass spectrometry (LC-MS) in conjunction with fragmentation studies (MS/MS). The rigid steroid skeleton and variations in double-bond position, epimerization, and hydroxyl/oxo substitution make differentiation and characterization by these platforms difficult.^15^ These challenges are further compounded by matrix effects and the low concentration of steroids in biological samples, requiring high sensitivity and, in doping contexts, trace quantification.^16^

Alternative gas-phase techniques are emerging as robust methods to support the characterization of metabolites.^9^ Ion mobility (IM), which separates ions based on their interactions with a buffer gas in the presence of an electric field, can measure differences in the shape and size of molecules.^17–20^ This is realized through instrument-independent collision cross section (CCS) values. However, structurally similar molecules such as those studied here often only yield small differences in CCS values and, when present in mixtures, are not distinguished easily. The advent of high-resolution ion mobility techniques such as cyclic IM, structures for lossless ion manipulations (SLIM), and trapped ion mobility spectrometry (TIMS) can combat these shortcomings.^21–24^ While the resolving power of traditional traveling wave IM operates between 40 and 50, high-resolution IM instruments can offer resolving powers of up to one magnitude higher.^25^ High-resolution IM has been applied to aid the separation of metabolites,^8^ glycans,^26^ and lipids,^27^ adding a dimension of separation for closely related species.

Gas-phase infrared (IR) spectroscopy is a complementary technique which can offer isomer-specific vibrational fingerprints for a range of biomolecules, including glycans,^28–30^ proteins,^31^ lipids,^32^ nucleotides,^33^ and metabolites.^9,34^ Ions are irradiated with IR light, and when the wavelength is in resonance with the vibrational modes of the ion, it is excited, resulting in a measurable system response. To limit spectral congestion and improve resolution, gas-phase IR spectroscopy can be conducted at cryogenic temperatures. A particularly powerful approach relies on ion encapsulation in ultracold helium nanodroplets, which evaporate upon IR excitation and release the bare ions, which in turn are detected with mass spectrometry.^35^ The elucidation of gas-phase structures can be attained through the comparison of experimental IR spectra to those computationally produced from vibrational frequencies based on density functional theory (DFT) calculations.^36,37^

Although less common in metabolite analysis, IM and gas-phase IR spectroscopy have been used to characterize the metabolome,^38^ with libraries containing CCS values of up to 300 steroids and steroid esters compiled.^39,40^ Analysis of steroid isomers by IM has shown successful discrimination, aided by the investigation of dimers,^41^ derivatized ions,^42,43^ and alternative adducts,^44–46^ as well as by using different drift gases.^41,47^ High-resolution ion mobility has allowed more complete separation of steroids,^48^ often when complexed to other molecules such as cyclodextrins.^49,50^ However, baseline separation of similar isomers, particularly as protonated species, remains difficult, and information on their gas-phase structures remains scarce.^47^

The isomeric corticosteroids, aldosterone and cortisone, differ only in the positions of functional groups; however, this leads to vastly different biological roles. Aldosterone controls salt and water balance, whereas cortisone is biologically inactive and readily converted to cortisol.^51^ Previous analysis by LC-MS/MS has demonstrated separation between aldosterone and cortisone.^52,53^ Although IM separation has been observed,^41^ features of monomeric, dimeric, and trimeric metal adducts are often reported as overlapping.^44,54^ For aldosterone, multiple features have been found with IM, indicating the possible presence of multiple gas-phase conformers.^54^

Hydroxyprogesterones (OHPs) 11β-OHP, 17α-OHP, and 21-OHP differ only by the position of the hydroxyl group within the steroid skeleton. OHPs hold key roles in reproductive processes^55^ and adrenal function,^56^ where 17α-OHP is a biomarker for congenital adrenal hyperplasia and thus requires specific testing.^57^ Despite the similarity of fragments, analysis by LC-MS/MS has separated the isomers,^52,53^ with optimized solvent conditions, longer retention times, and derivatization increasing separation.^58,59^ A study to determine the interference of isomers with the detection of 17α-OHP, however, found that not all laboratories could distinguish isomers, and approximately half did not consider all possible isobars.^60^ Partial separation by IM has been realized with derivatization,^43^ and by investigating silver adducts with cyclic IM,^23,45^ however, in both studies, the three isomers could not be baseline separated. High-resolution IM has successfully separated 17α-OHP and 21-OHP when complexed to form heterodimers with a steroid analogue.^50^

For both sets of isomers, no gas-phase IR spectroscopy studies have been reported. This technique provides additional insights into gas-phase structures such as protonation sites^61^ and evidence for gas-phase tautomerism,^9^ both of which are potentially relevant for steroid structures. The importance has been demonstrated in the analysis of testosterone, where it was not possible to determine protonation site due to rearrangements and common fragment structures.^62,63^ For applications in metabolomics, the adoption of cryogenic gas-phase IR spectroscopy integrated into commercial instrumentation has great potential in the identification of unknown metabolites and gas-phase structures.^64^ Particularly, the combination of separation by chromatography and/or IM and characterization by IR is increasingly used and can be applied for the structural elucidation of molecules from complex mixtures.^65,66^

Here, we apply ion mobility mass spectrometry (IM-MS) and cryogenic gas-phase IR spectroscopy to distinguish and characterize corticosteroids and hydroxyprogesterones (Figure 1). We find that although both sets of isomers remain challenging to separate by IM as protonated species, they exhibit distinct IR spectra. Through comparison to DFT-calculated theoretical IR spectra, we assign gas-phase structures, indicating a preference for protonation at the C3 carbonyl group. We further emphasize how the spectral range between 1700 and 1800 cm^–1^ is highly diagnostic for distinguishing the steroid isomers. Of particular interest is the absence of any band in this region for aldosterone, suggesting the formation of a bicyclic structure. Overall, our results highlight the potential of gas-phase IR spectroscopy as a powerful platform for isomer-specific analysis in metabolomics and metabolite profiling.

**Figure 1:**
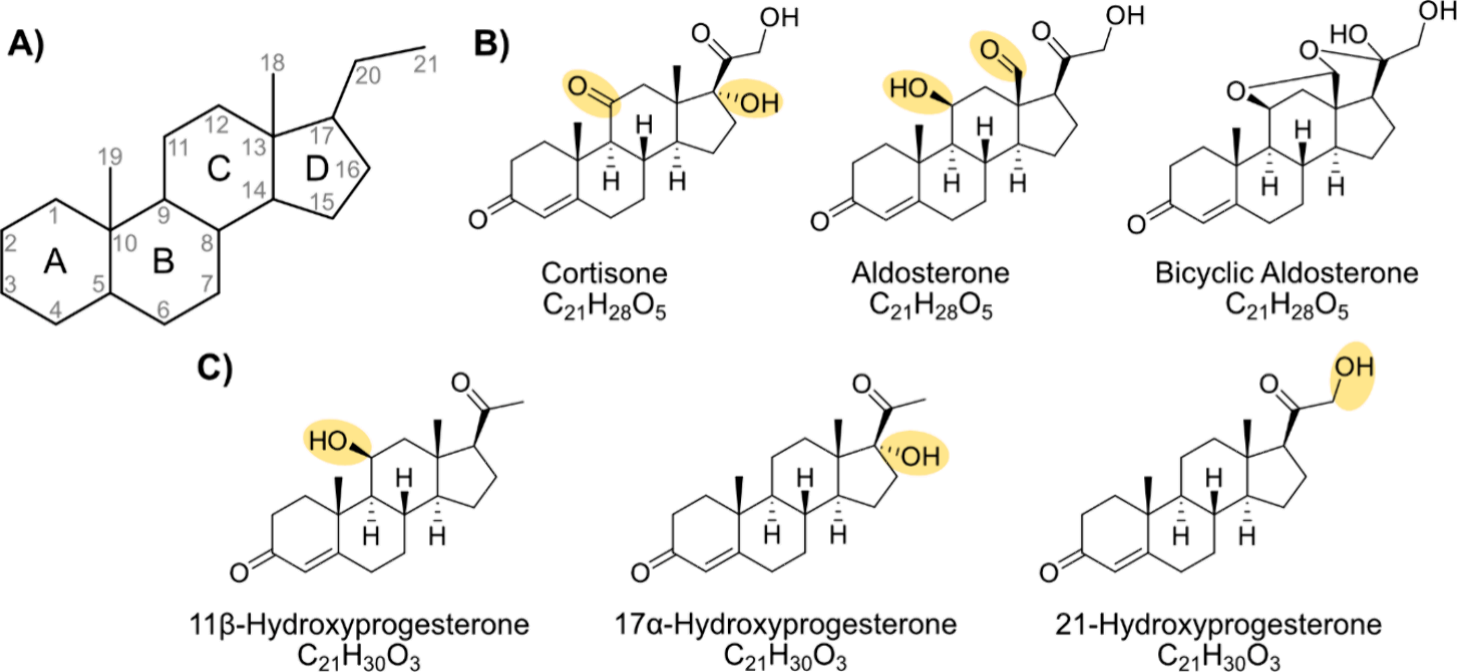
(A) Core structure of the steroids containing four rings with carbon numbers added. (B) Structures of aldosterone and cortisone with their respective formulas and the bicyclic structure of aldosterone. (C) Structures of the three hydroxyprogesterones with their respective formulas. Differences in the structures of isomers are highlighted.

## Methods

Aldosterone and 11β-OHP standards were obtained from Merck. Cortisone, 17α-OHP, and 21-OHP were obtained from TCI chemicals. For IM-MS analysis, 10 μM solutions of individual standards and mixtures of the isomeric sets were prepared in 1:1 MeOH:H_2_O (v/v) with 0.1% FA. For gas-phase IR experiments, the solutions were prepared as 250 μM in 1:1 MeOH:H_2_O (v/v) with 0.1% FA. Samples were transferred to the gas phase using nanoelectrospray ionization (nESI) from capillaries with an inner tip diameter of 1–2 μm, home-pulled glass on a pipette puller (Model: P-2000; Sutter Instrument Company).

For IM-MS measurements, a Synapt G2-S platform was used in the positive ionization mode. A potential of 0.8 kV was applied through the platinum wire inserted into the capillary tip. The source temperature was set at 30 °C, the cone voltage was at 20 V, and the source offset was at 5 V. For well-resolved IM settings, a mass range of *m*/*z* 50–600 was used with a traveling wave velocity of 1150 m/s and a traveling wave height of 40 V applied in the IMS cell. For experimental ^TW^CCS_N2_ values, a mass range of *m*/*z* 50–3000 was used, with a traveling wave velocity of 600 m/s and traveling wave height of 40 V in the IMS cell. The arrival times were converted into instrument-independent ^TW^CCS_N2_ values using established calibration procedures^67^ with Agilent tune mix as a calibrant.^68^

Helium nanodroplet gas-phase IR spectroscopy was conducted on our home-built instrument described in detail previously.^69,70^ A capillary voltage of 0.8–1.0 kV was applied through a platinum wire. The protonated ions were *m*/*z*-selected in a quadrupole and transferred to a hexapole trap where they were cooled to 90 K via collisions with helium gas. Superfluid helium nanodroplets (0.37 K), generated by a pulsed Even–Lavie valve,^71^ then picked up the trapped ions. These were transported to the interaction region where they were irradiated with an IR beam generated by the Fritz Haber Institute free-electron laser (FHI-FEL) operated in the wavenumber range *ṽ* = 800–1800 cm^–1^.^72^ The excitation of the steroid cations with resonant photons led to their release from the helium nanodroplets, which were then detected with a time-of-flight mass analyzer. The IR spectra of the ions of interest were obtained by plotting ion intensity against wavenumber. Each spectrum was measured twice, and an average spectrum is presented here. Due to a technical issue with the FEL, the region of 1650–1800 cm^–1^ had to be remeasured on a separate occasion. Spectra have all been normalized to account for this, with a break in the *x*-axes indicating the different origins of the spectra parts.

Experimental IR spectra were compared to those based on DFT calculations.^73^ For all steroid cations, each candidate protomer was subjected to a conformational search using CREST software.^74^ The 30 lowest energy conformers were reoptimized in Gaussian 16 ^75^ at the PBE0+D3/6-311+G­(d,p)^76,77^ level. Tautomer structures were further sampled directly through CREST and equally reoptimized with Gaussian 16. All candidates were ranked by their free energy at 90 K. Harmonic frequencies were computed, and the obtained IR spectra were normalized and scaled by an empirical factor of 0.965.^78^ For the two assigned main species of each steroid protomer, theoretical CCS values were obtained from IMoS software (^TH^CCS_N2_, TH = theoretical), using the trajectory method in nitrogen gas with quadrupole potential at room temperature (number of orientations = 3, gas molecules per orientation = 300,000).^79^

## Results

### Ion Mobility

All five steroids were transferred to the gas phase as protonated cations, and their individual ^TW^CCS_N2_ distributions were recorded (Figure 2 and Table S1 for the experimental and theoretical CCS_N2_ values). The structural similarity of the steroids is reflected in the small ^TW^CCS_N2_ differences observed. Aldosterone is found to have a smaller CCS value than cortisone, and for the OHPs, 11β-OHP is marginally smaller than 17α-OHP, with 21-OHP having the largest CCS value of the three isomers. Even for 11β-OHP and 21-OHP, which show the largest difference, the variation remains below 2%. Baseline separation of peaks with such small differences would require resolving powers >100, exceeding the capabilities of the instrument used here, but potentially achievable by higher resolution platforms.^80–82^ Comparable CCS variations (1.5–2%) have been reported for steroid ions across different instruments in a comparison study, indicating that interplatform variability is comparable to differences between the isomers.^40^ Consistent with this, no distinct IM peaks were observed for either set of isomers when analyzed as mixtures (Figure S1), reflecting both the minimal CCS differences and the limited resolving power of the instrument.

**Figure 2:**
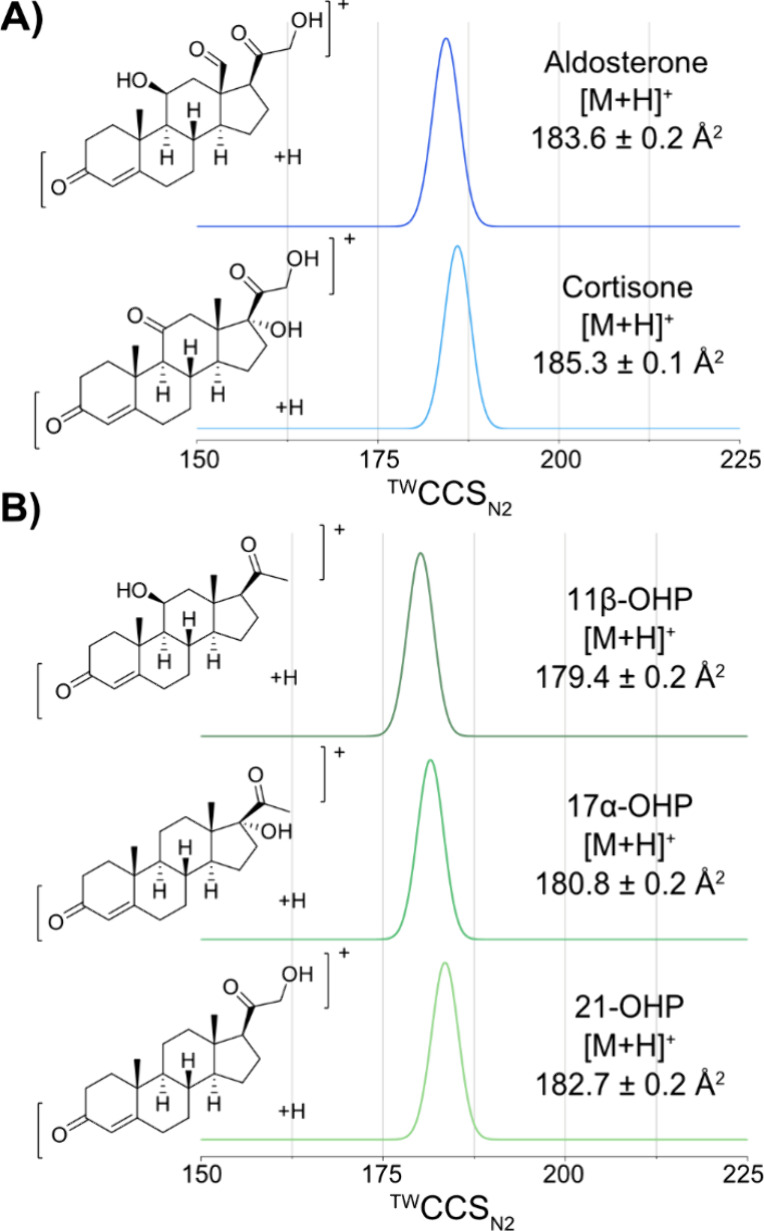
^TW^CCS_N2_ distributions of the steroid standards: (A) corticosteroids, aldosterone and cortisone; (B) hydroxyprogesterones, 11β-OHP, 17α-OHP, and 21-OHP. For both sets of isomers, only small differences in the ^TW^CCS_N2_ distributions were found.

### Cryogenic Gas-Phase Infrared Spectroscopy

The protonated ions of the steroids were characterized using cryogenic gas-phase infrared spectroscopy, and the mass spectra from the instrument were acquired and displayed in Figures S2–S6. The IR spectra were measured from 800 to 1800 cm^–1^. The fingerprint region (800–1400 cm^–1^) contains C–O and C–C stretching and the O–H and C–H bending vibrations, whereas the functional group region (1400–1800 cm^–1^) is dominated by C=O and C=C stretching vibrations. The fingerprint region can yield complex spectra, and thus the functional group region is often preferred for assignments.^29^ The IR spectra of aldosterone were measured at different macropulse energies, and a macropulse energy of 20 mJ was chosen for irradiation of all steroid protomers. Structures were assigned by comparison with the calculated IR spectra. These spectra are generated with protonation sites manually sampled at all carbonyl groups, shown in Figure 3. It was also observed that the orientation of C3 protonation yielded distinct spectra.

**Figure 3:**
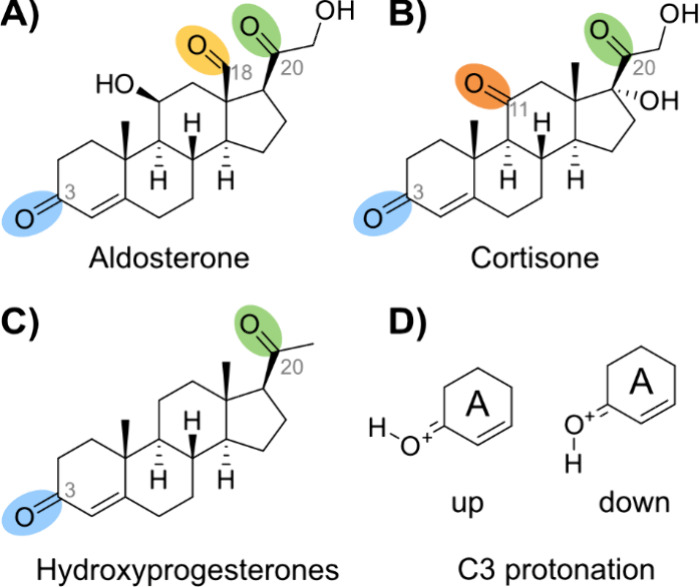
Protonation sites of the steroids (A) aldosterone, (B) cortisone, and (C) the hydroxyprogesterones. C3 protonation is highlighted in blue, C20 in green, C18 in yellow, and C11 in orange. (D) displays the orientation of protonation at C3.

### Corticosteroids: Aldosterone and Cortisone

For aldosterone, the experimentally measured IR spectrum showed the most intense bands in the region between 1450 and 1600 cm^–1^, with no features present beyond 1600 cm^–1^. This indicates the absence of C=O stretching vibrations and hence carbonyl groups as well as suggesting that the C4=C5 bond is conjugated. Protonation sites were modeled at the three carbonyl groups and sampled together with potential tautomer structures. One particular tautomeric candidate structure, known from the solid phase, is a bicyclic acetal, which forms through nucleophilic attacks of the C11 hydroxyl group on the C18 aldehyde, and subsequently of the C18 hydroxyl group on the C20 ketone.^83^ This results in the formation of two new five-membered rings (Figure 1B).

Experimental bands were assigned by comparison with DFT-calculated theoretical spectra, where protonation at the C3 carbonyl group yielded the lowest-energy theoretical candidates. The best agreement was found with the two energetically lowest C3 protomers of the bicyclic structure (Figure 4: C3down_2ring and C3up_2ring), which only differ by the orientation of protonation (Figures 3D and S7), resulting in the observed spectral differences between 1450 and 1600 cm^–1^. The major peaks at 1500 cm^–1^ and the broad band at 1550 cm^–1^ can be assigned as conjugations of the C3–C5 π-system following protonation at C3 carbonyl. The similarity to the experimental spectrum and the calculated structures suggests that both conformers are present simultaneously. Other candidate structures that only form one or no intramolecular rings can be excluded, as no band was found experimentally beyond 1700 cm^–1^ (Figure 4). All sampled structures and energies are displayed in Figure S7 and Table S2.

**Figure 4:**
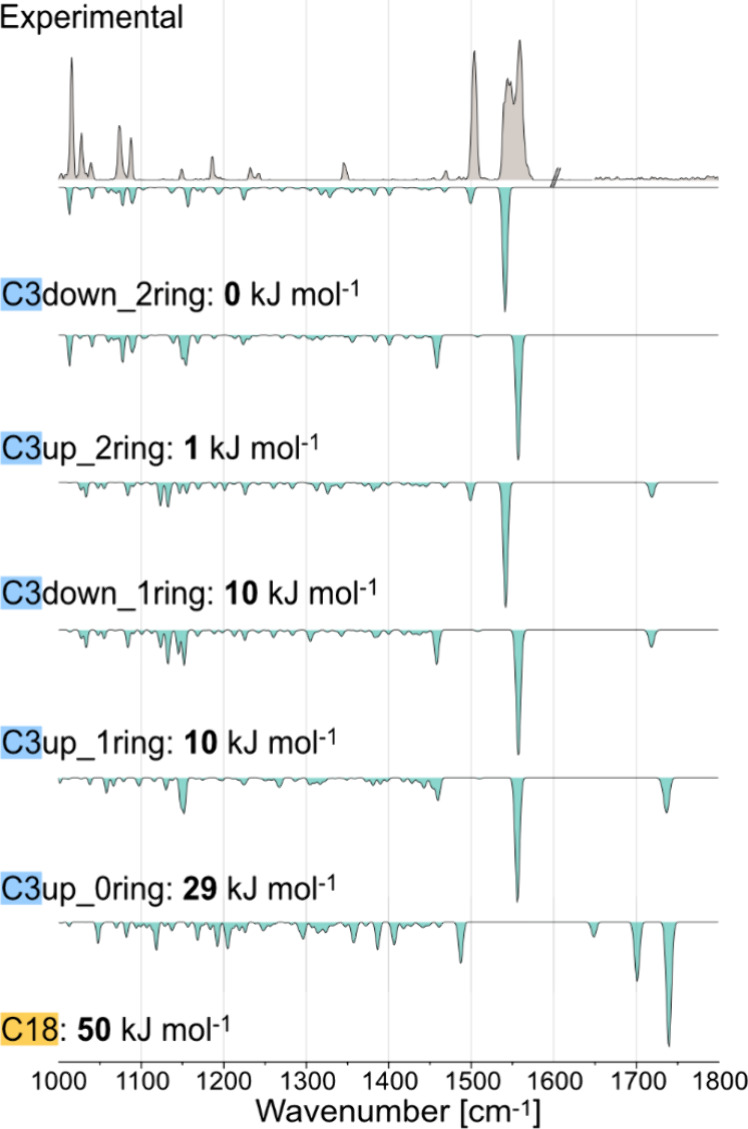
Cryogenic infrared spectra of protonated aldosterone at 20 mJ FEL macropulse energy (gray). The calculated IR spectra are shown as inverted traces, where up and down refers to the orientation of the proton on the C3 carbonyl group and rings refers to the number of intramolecular rings formed. The experimental spectra are best matched to a mixture of structures C3down_2ring and C3up_2ring.

**Figure 5:**
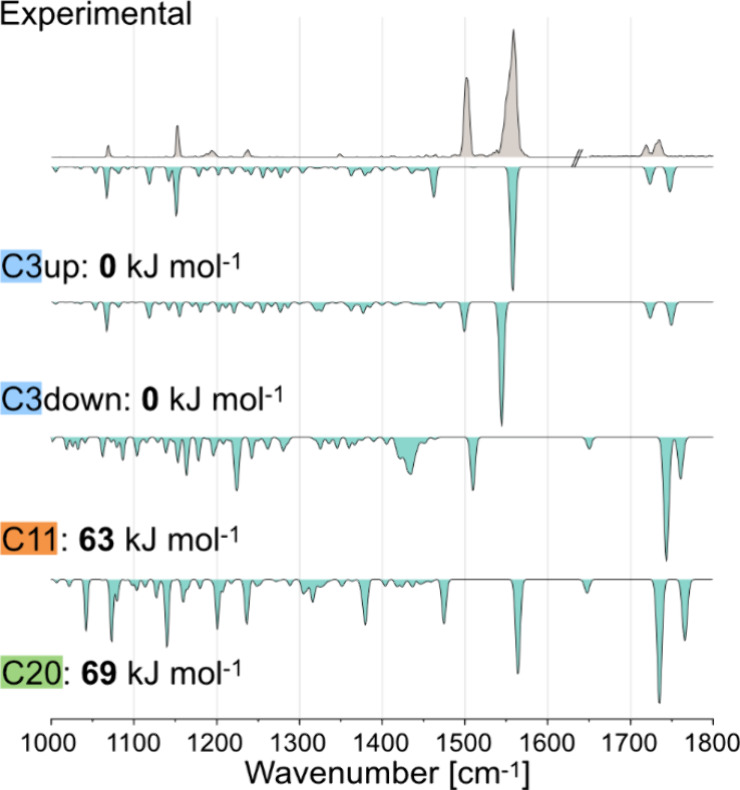
Cryogenic infrared spectra of protonated cortisone at 20 mJ FEL macropulse energy (gray). The calculated IR spectra are shown as inverted traces. C3up and C3down are low energy structures protonated at C3; also shown are the lowest energy structures protonated at C11 and at C20. The experimental trace can best be assigned to the two structures protonated at the C3.

The isomeric cortisone shares similar experimental features to aldosterone in the 1450–1600 cm^–1^ range, with the additional presence of bands in the C=O stretching region at 1719 and 1734 cm^–1^. The arrangement of the aldehyde and carbonyl groups in cortisone suggests that it is unlikely to form intramolecular rings due to distances and orientation, compounded by the rigidity of the steroid frame. Comparison to theoretically simulated spectra again suggests the preference for C3 carbonyl protonation. The two most stable C3 protonated structures are shown in Figure 5 (C3up and C3down). In combination, they correlate well with the experimental data, suggesting the presence of a mixture. The two bands beyond 1700 cm^–1^ can be assigned as carbonyl stretching at C17 and C20, inferring that no intramolecular ring formation has occurred. The structures following protonation at C11 and C20 carbonyl (Figure 5) are of much higher energy and do not agree with the experimental spectrum, suggesting that C3 protonated structures are present exclusively (Figure S8 and Table S3 for all candidate structures with energetics).

### Hydroxyprogesterones: 11β-OHP, 17α-OHP, and 21-OHP

Experimental spectra for the hydroxyprogesterone structures exhibit similar dominant bands in the 1450–1600 cm^–1^ region, with each isomer also displaying at least one band in the C=O stretching region beyond 1700 cm^–1^. Theoretical structures were modeled for protonation at both carbonyl groups. Similarly, tautomers were considered for possible keto–enol tautomerism. For each OHP, the lowest energy calculated structures were protonated at C3 and conjugated between C3 and C5. This agrees with the absence of an isolated C=C band in the experimental IR spectra. The multiple peaks observed in the 1550 cm^–1^ band again suggest different orientations of protonation at the C3 carbonyl group.

For 11β-OHP (Figure 6A), the best agreement was found with a mixture of two conformers of C3 protomers (Figure S9). From these structures, we can assign the 1730 cm^–1^ band as the C20=O stretching vibration and the major bands in the 1450–1600 cm^–1^ region as the ring A protonation and conjugation. For 17α-OHP (Figure 6B), the best agreement was again found with low energy C3 carbonyl protomers (Figure S10). Dominant experimental bands were assigned to the C3 conjugated system, and 1717 cm^–1^ was assigned as the C20=O stretching vibration. The combination of these conformers provides a good match with experimental data. For 21-OHP (Figure 6C), the assignment of the dominant bands remains the same (two conformers of the C3 protonated species; Figure S11) where the orientation of the proton at the C3 carbonyl results in distinct spectra and yields the broad band between 1540 and 1560 cm^–1^. The peak at 1732 cm^–1^ corresponds to the C20=O stretching vibration and contains two conformers (Figure S11), which likely arise from the orientation of the alkyl chain from C17 and the proton on C21. For all three structures, the lack of similarity to the C20 protonated candidates infers that only C3 protonation occurs. The structures and energies are shown in Figures S9–S11 and Tables S4–S6.

**Figure 6:**
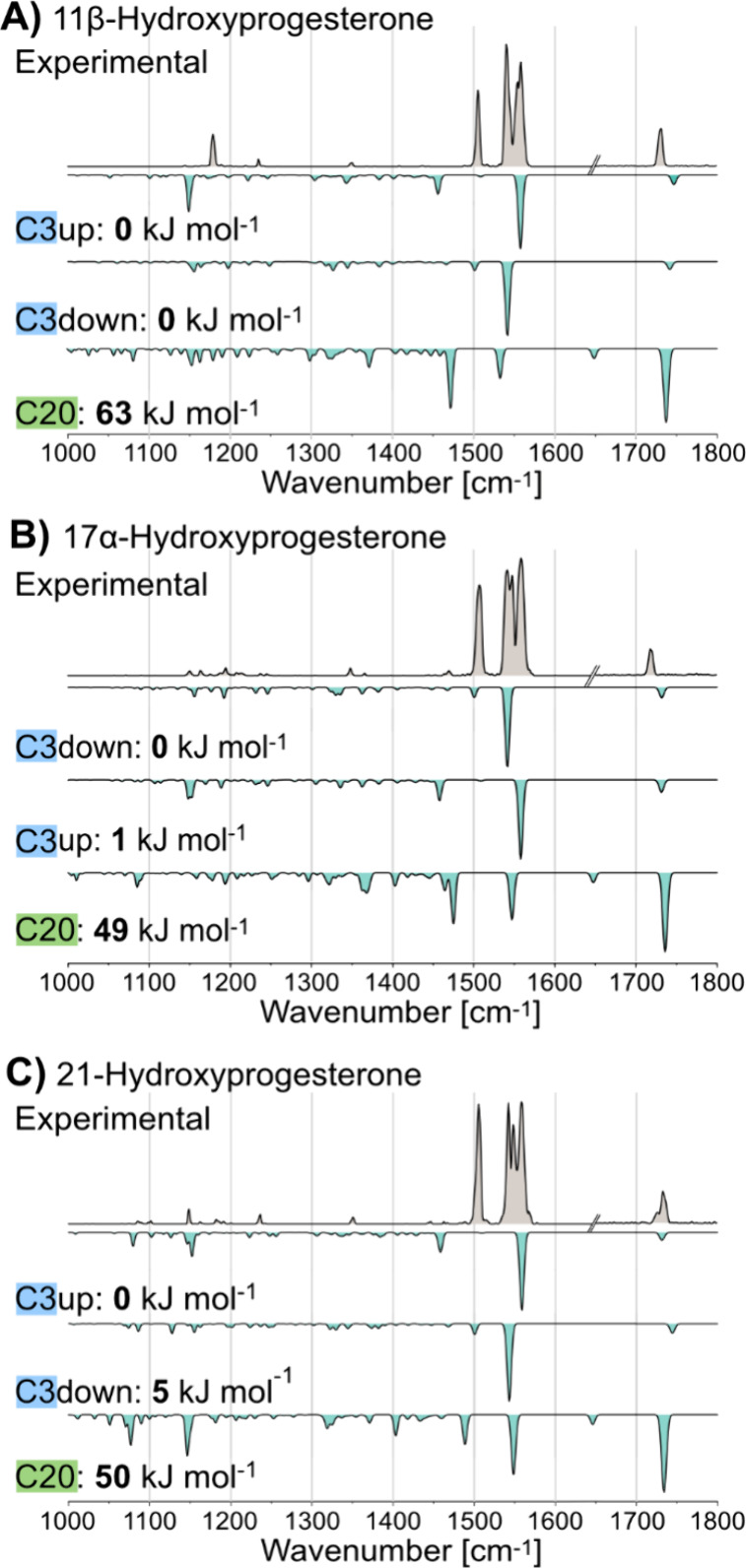
Cryogenic infrared spectra of the protonated cations for (A) 11β-OHP, (B) 17α-OHP, and (C) 21-OHP (gray traces). Computed spectra are shown as inverted traces. For all species, the best agreement was found with C3 carbonyl protonation.

### Theoretical CCS_N2_ Calculations of Assigned Candidate Structures

For each protonated steroid cation, theoretical CCS_N2_ values were calculated for the two main contributing assigned candidates. This was realized using the trajectory method including quadrupole potential, as implemented in IMoS (Table S1).^79^ The absolute values agree well with the respective experimental values (maximum deviation of 1.6%), and the relative trends were also reproduced. Both the experimental ^TW^CCS_N2_ and theoretical ^TH^CCS_N2_ values reflect the more compact cyclized aldosterone compared to the open cortisone structure. For the OHPs, 21-OHP yielding the largest CCS_N2_ likely arises from the extended hydroxy branch at C21. For 11β-OHP and 17α-OHP, both ^TW^CCS_N2_ and ^TH^CCS_N2_ values are closer, meaning that differences in shape are more subtle. Together, the theoretical ^TH^CCS_N2_ values support the gas-phase IR-spectroscopy-based assignment of the steroid cations.

## Discussion

Isobaric steroid structures differ greatly in their biological roles, making reliable isomer separation and identification essential.^2^ This is particularly crucial where multiple isomers coexist, and only specific structures serve as biomarkers of disease or performance-enhancing agents. Here, for example, 17α-OHP is the only isomer to serve as a diagnostic biomarker for 21-hydroxylase deficiency, linked to congenital adrenal hyperplasia.^57^ For the two isomeric sets studied here, we observe that the structural changes result in only minimally different ^TW^CCS_N2_ and ^TH^CCS_N2_ values. In a mixture, the individual species are not separable with the instrument platform deployed, posing a challenge that has been observed previously.^44,45^ IM instruments with higher resolving powers have the possibility of separating the protonated species, but remain limited in the direct structural information they provide. The use of a spectroscopic dimension can aid structural identification, and in this case, multiple ion populations were found for all steroids that differ only in the orientation of the proton. This is not observed by IM, and although the instrument used here has limited resolution, the calculated ^TH^CCS_N2_ values for up/down protonation at most deviate by 0.4%, making differentiation challenging even with high-resolution IM. Due to their small energetic differences and the presence of both orientations, it is plausible that these species interconvert and appear as a single ion population.

The ability of gas-phase IR spectroscopy to characterize isomeric metabolites is highly valuable in omics fields, where thorough identification following separation from complex mixtures, is crucial.^65^ This has been demonstrated by the Oomens group, where gas-phase IR spectroscopy was used to identify a biomarker from other possible isomers (*N*-acetylhexosamines) after *m*/*z*-isolation of the ion.^34^ In this work, gas-phase IR spectroscopy can discriminate protonated steroid ions through diagnostic features in the region between 1700 and 1800 cm^–1^ (Figure 7), making this technique potentially suitable for metabolomics workflows. The analytical value lies in the comparison of experimental IR spectra with reference databases containing IR data, allowing unambiguous identification as previously realized for glycans.^84^ The use of IM (including ultrahigh resolution IM) combined with IR can also be exploited for small molecules, where isomeric structures are separated by IM and characterized by IR.^65,85^

**Figure 7:**
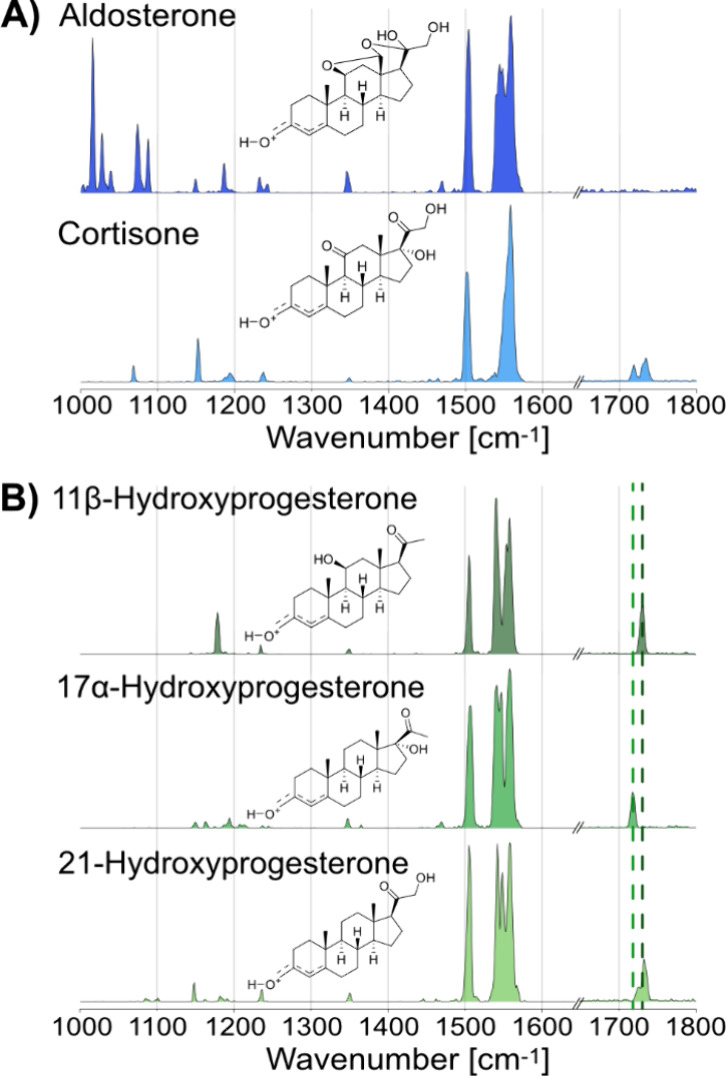
Cryogenic infrared spectra and assigned structures of the protonated cations for (A) corticosteroids (aldosterone and cortisone, *m*/*z* 361) and (B) hydroxyprogesterones (11β-OHP, 17α-OHP, and 21-OHP, *m*/*z* 331). The isomers can be distinguished by their gas-phase IR spectra in the region between 1700 and 1800 cm^–1^. For the two corticosteroids, the formation of intramolecular rings in aldosterone leads to distinct changes in the IR spectrum, whereas for the hydroxyprogesterones, the number and positions of the C=O stretching vibrations vary, as indicated by dashed lines.

We further provide the first gas-phase characterization of steroid ions, which is fundamentally important to understand their MS-based separation and analysis approaches. The presence of multiple protonation sites and functional groups that potentially form keto–enol tautomers leads to many possible structural candidates for steroids, which cannot easily be elucidated by traditional MS-based techniques. Different protomers can also yield different fragmentation behavior and mobility, meaning that the understanding of protomer site and structure is important for accurate interpretation and annotation.^8^

Particularly interesting is the example of aldosterone, which exists in an equilibrium of the bicyclic and cyclic (11–18 hemiacetal) form in aprotic solvents,^86,87^ and in crystal form as the bicyclic structure (Figure 1B).^83^ Gas-phase measurements are often regarded as similar to solution studies in aprotic solvents due to similar dielectricity constants. This suggests that a bicyclic structure is also plausible in vacuo, and we indeed observed this type of intramolecular self-stabilization. DFT-based energetics suggest that the formation of intramolecular rings are energetically favorable (Table S2), with structures not involving ring formation being ca. 30 kJ mol^–1^ higher in energy. This stable gas-phase structure may parallel the biologically active form, as the cyclic form has been recognized to contribute to its unique mineralocorticoid specificity (where the geometry adopted by the cyclic structure fits the receptor).^88^ Cortisone, however, lacks the C18 carbonyl and hence retains the open framework and C=O stretching vibrations in the IR spectrum. This unmodified network reflects a more flexible and polarizable scaffold, possibly correlated with its reduced bioactivity,^89^ as well as higher accessibility for enzymatic reductions.^90,91^ Together, this indicates that the methods implemented here can mimic biological relevance in the gas phase.^92^

The preference for protonation at the C3 position for all steroids suggests that this is the most basic site in vacuo. This can be rationalized by stabilization of the positive charge through the conjugation within ring A (Figure 1A), while the increased accessibility of the C3 carbonyl may further promote the protonation. For all steroid cations, the lowest energy conformers calculated were found experimentally, with no other protomers observed. This agrees well with the DFT energies of the other protomers, which are at least 49 kJ mol^–1^ higher than those of C3 protonation. The use of protic solvents is also expected to help conversion to the more thermodynamically stable protomer, even though comparison of aprotic and protic solvents has shown a negligible difference in IM distributions of steroids.^61,50^ For these reasons, it can be assumed that structures observed are in thermodynamic equilibrium, although kinetic trapping cannot be fully excluded.^61^ This localization of the charge at the C3 position could be a contributing factor to the challenge of distinguishing these metabolites, as different protomers can show separation via other techniques. For example, protomers of small molecules have previously been distinguished in infrared multiple photon dissociation (IRMPD) spectra,^61,93^ and by ion mobility, including differential IM,^94^ cyclic IM,^8^ and TIMS.^95^

We further did not observe any keto–enol tautomerism, although such tautomers of other small molecule classes have been resolved by IM^8,96^ and gas-phase IR spectroscopy.^9^ Sampled candidate structures with this type of tautomerism were scarce and consistently at higher energies (27–54 kJ mol^–1^). This is likely due to the high stability of the C=O bond and/or the lack of stabilization of the O–H enol in the absence of protic solvents. For other metabolites, the enol form has been observed in the gas phase, especially when stabilized by intramolecular hydrogen bonding.^97^ For steroids, not only is the keto form intrinsically more stable, but also the rigidity of the ring system and restricted conformational freedom prevent any stabilization through intramolecular H-bonding or conjugation.

## Conclusions

We demonstrate that cryogenic gas-phase IR spectroscopy can distinguish two sets of isomeric steroid structures, namely, corticosteroids and hydroxyprogesterones, largely through the number and position of carbonyl stretching vibrations in the region between 1700–1800 cm^–1^. This is particularly relevant as steroid structures are often highly similar and challenging to differentiate by other MS-based techniques, including IM. We further provide the first experimental evidence for steroid structures in the gas phase, elucidating the preferred protonation site at the C3 carbonyl as well as the absence of keto–enol tautomerism. For aldosterone, we found that the bicyclic structure, previously found in solution and in the crystal form, retains this conformer in the gas phase. Taken together, gas-phase IR spectroscopy offers a complementary technique for the structural elucidation of steroids and their fingerprinting for steroid omics workflows.

## Supplementary Material



## Data Availability

Supporting data referred to in this manuscript is contained within a Supporting Information document and in a supplementary data set available on Figshare (DOI: 10.6084/m9.figshare.30849650).

## Notes

^TW^CCS_N2_ values obtained for the protonated steroid ions and theoretically (TH) calculated ^TH^CCS_N2_ values (Table S1), energetics of protonated steroid structures (Tables S2–S5), arrival time distributions of individual steroids and mixtures of the isomers (Figure S1), mass spectra of the protonated steroids (Figures S2−S6) and low energy structures of the steroids (Figures S7−S11) (PDF)
