# Supplementary material for: Steroid Fingerprinting with Cryogenic Gas-Phase Infrared Spectroscopy
Source: ACS Meas Sci Au. 2026 Apr 13;6(3):765–74. doi: 10.1021/acsmeasuresciau.6c00043 (PMC13281185; doi:10.1021/acsmeasuresciau.6c00043)
Supplement: Supplementary file 1 [file tg6c00043_si_001.pdf]

Supporting Information

Steroid Fingerprinting with Cryogenic Gas-Phase Infrared Spectroscopy

Caitlin Walton-Doyle[1,2,*], Gurpur Rakesh D. Prabhu[1,2], Niklas Geue[1,2], Gerard Meijer[2], Gert von Helden[2] and Kevin Pagel[1,2,*]

[1]Institute of Chemistry and Biochemistry, Freie Universität Berlin, Altensteinstraße 23a, 14195 Berlin, Germany. [2]Department of Molecular Physics, Fritz-Haber-Institut der Max-Planck-Gesellschaft, Faradayweg 4–6, 14195 Berlin, Germany.

[*]Corresponding Author: caitlin.walton-doyle@fu-berlin.de, kevin.pagel@fu-berlin.de

Table of Contents

Table S1: $^{TW}CCS_{N2}$ values obtained for the protonated steroid ions and theoretically (TH) calculated $^{TH}CCS_{N2}$ values 3
Figure S1: Arrival time distributions of individual steroids and mixtures of the isomers.... 4
Figure S2: Mass spectrum of aldosterone 5
Figure S3: Mass spectrum of cortisone 6
Figure S4: Mass spectrum of 11β-hydroxyprogesterone........ 7
Figure S5: Mass spectrum of 17α-hydroxyprogesterone 8
Figure S6: Mass spectrum of 21-hydroxyprogesterone........ 9
Figure S7: Lowest energy DFT optimised structures of aldosterone. 10
Table S2: Energetics of protonated aldosterone structures 11
Figure S8: Lowest energy DFT optimised structures of cortisone... 13
Table S3: Energetics of protonated cortisone structures. 14
Figure S9: Lowest energy DFT optimised structures of 11β-hydroxyprogesterone.. 16
Table S4: Energetics of protonated 11β-hydroxyprogesterone structures........ 17
Figure S10: Lowest energy DFT optimised structures of 17α-hydroxyprogesterone... 19
Table S5: Energetics of protonated 17α-hydroxyprogesterone structures 20
Figure S11: Lowest energy DFT optimised structures of 21-hydroxyprogesterone. 22
Table S6: Energetics of protonated 21-hydroxyprogesterone structures 23

Table S1: $^{TW}CCS_{N2}$ values obtained for the protonated steroid ions and theoretically (TH) calculated $^{TH}CCS_{N2}$ values using the trajectory method in IMoS, both included experimental errors. The $^{TH}CCS_{N2}$ values were calculated for the two conformers assigned to be present in the gas-phase.

Structure	Experimental $^{TW}CCS_{N2}$ Value of $[M+H]^+$ in Å^2	Theoretical $^{TH}CCS_{N2}$ Value of $[M+H]^+$ in Å^2
Aldosterone	183.6 ± 0.2	C3down_2ring = 181.1 ± 0.8
		C3up_2ring = 181.7 ± 0.7
Cortisone	185.3 ± 0.1	C3up = 185.7 ± 1.3
		C3down = 186.3 ± 0.6
11β-Hydroxyprogesterone	179.4 ± 0.2	C3up = 177.1 ± 0.6
		C3down = 176.9 ± 0.5
17α-Hydroxyprogesterone	180.8 ± 0.2	C3down = 177.9 ± 0.9
		C3up = 178.2 ± 1.1
21-Hydroxyprogesterone	182.7 ± 0.2	C3up = 181.8 ± 0.5
		C3down = 182.5 ± 1.4

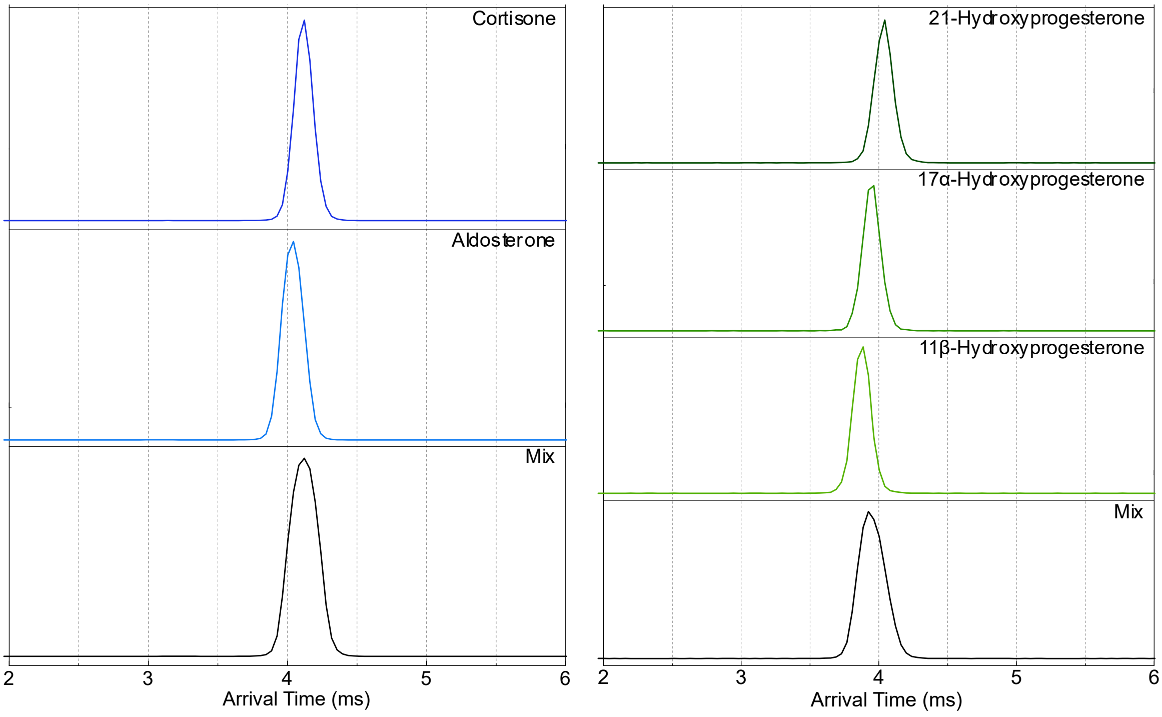

Figure S1: Arrival time distributions of individual steroids and mixtures of the isomers. For both sets, the mixtures show a unimodal distribution, indicating that the isomers cannot be resolved under the instrument conditions used.

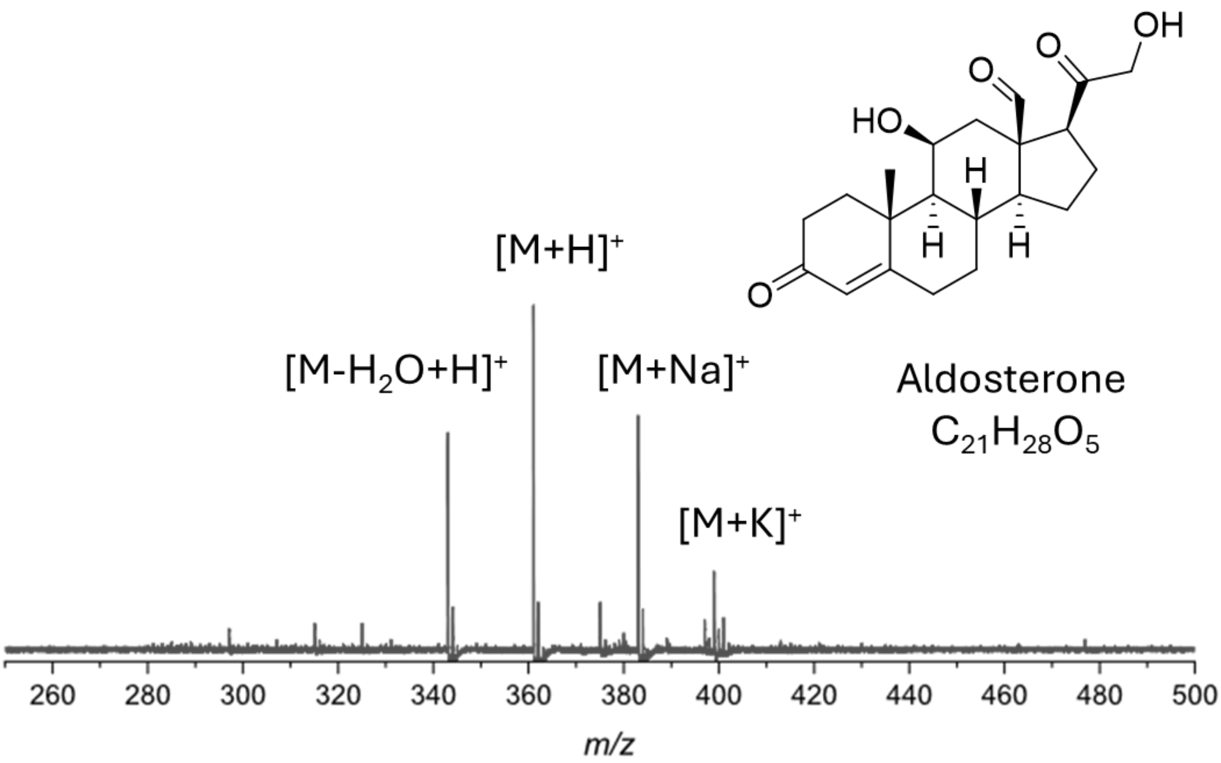

Figure S2: Mass spectrum of aldosterone in 1:1 methanol/water (v/v) with 0.1% formic acid (final concentration: 250 µM) when analysed on the cryogenic gas-phase IR-MS platform. Sodium and potassium adducts are also present.

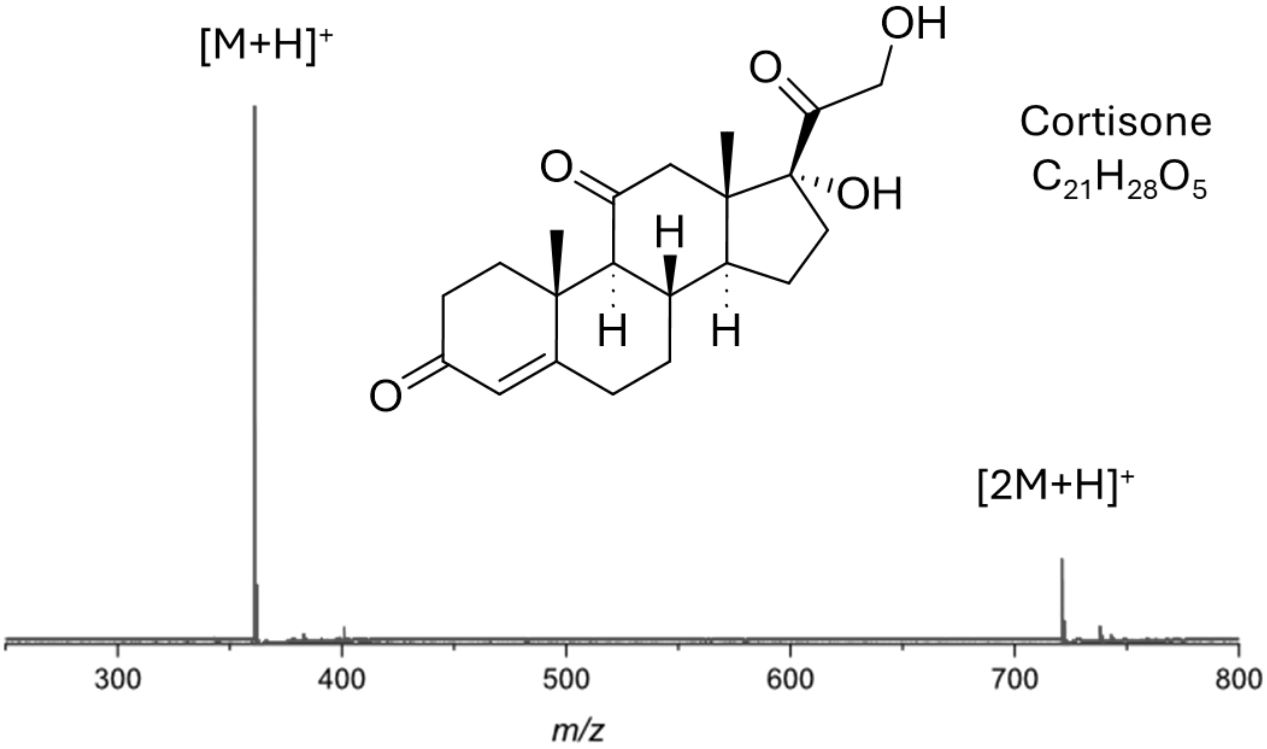

Figure S3: Mass spectrum of cortisone in 1:1 methanol/water (v/v) with 0.1% formic acid (final concentration: 250 μM) when analysed on the cryogenic gas-phase IR-MS platform. The protonated dimer is also present.

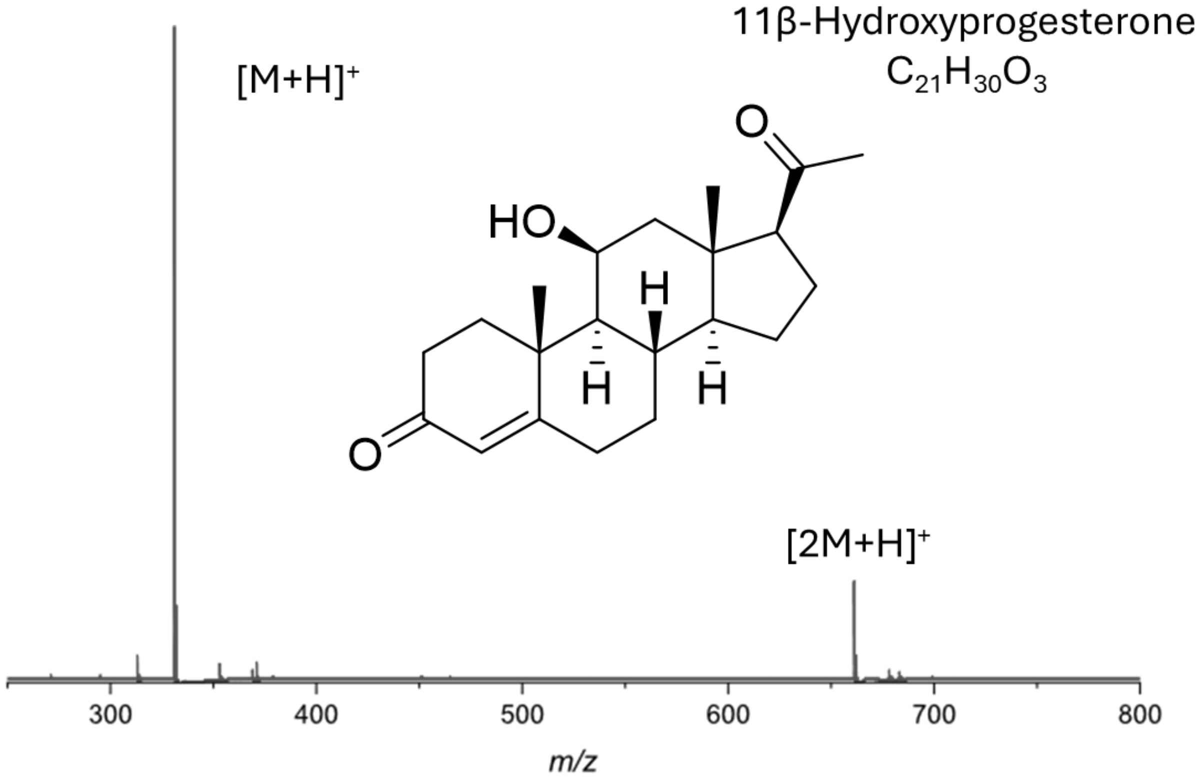

Figure S4: Mass spectrum of 11β-hydroxyprogesterone in 1:1 methanol/water (v/v) with 0.1% formic acid (final concentration: 250 μM) when analysed on the cryogenic gas-phase IR-MS platform. The protonated dimer is also present.

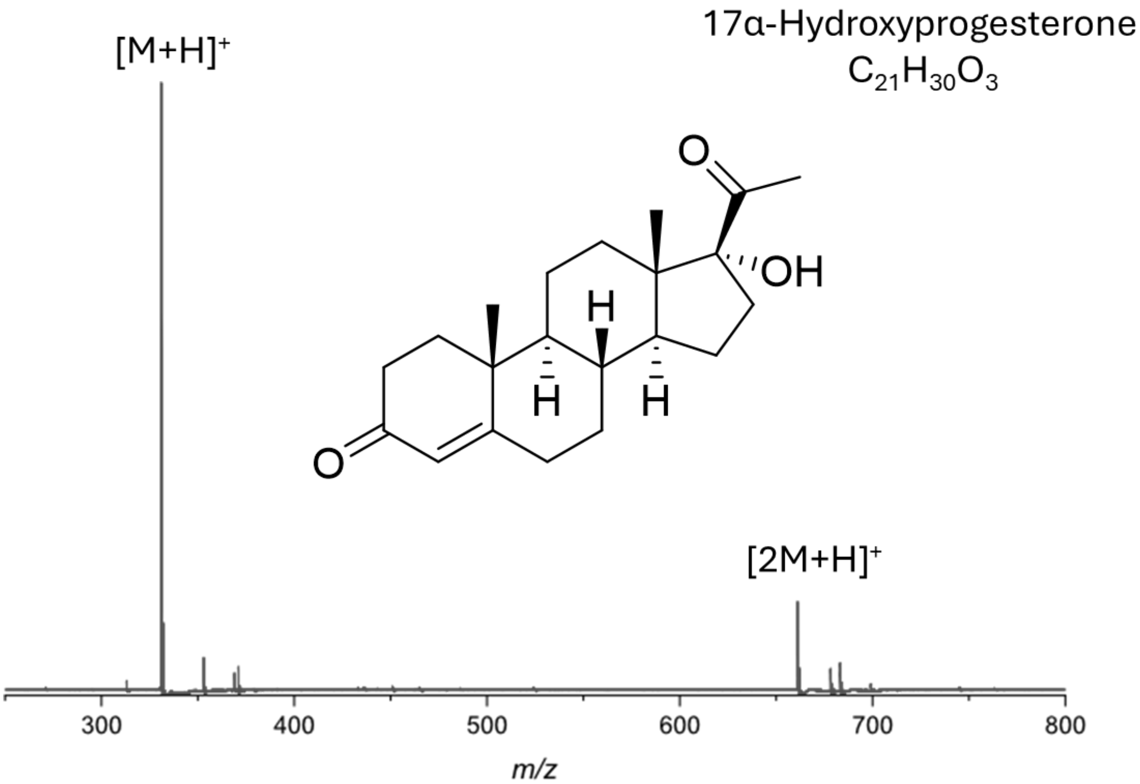

Figure S5: Mass spectrum of 17α-hydroxyprogesterone in in 1:1 methanol/water (v/v) with 0.1% formic acid (final concentration: 250 μM) when analysed on the cryogenic gas-phase IR-MS platform. The protonated dimer is also present.

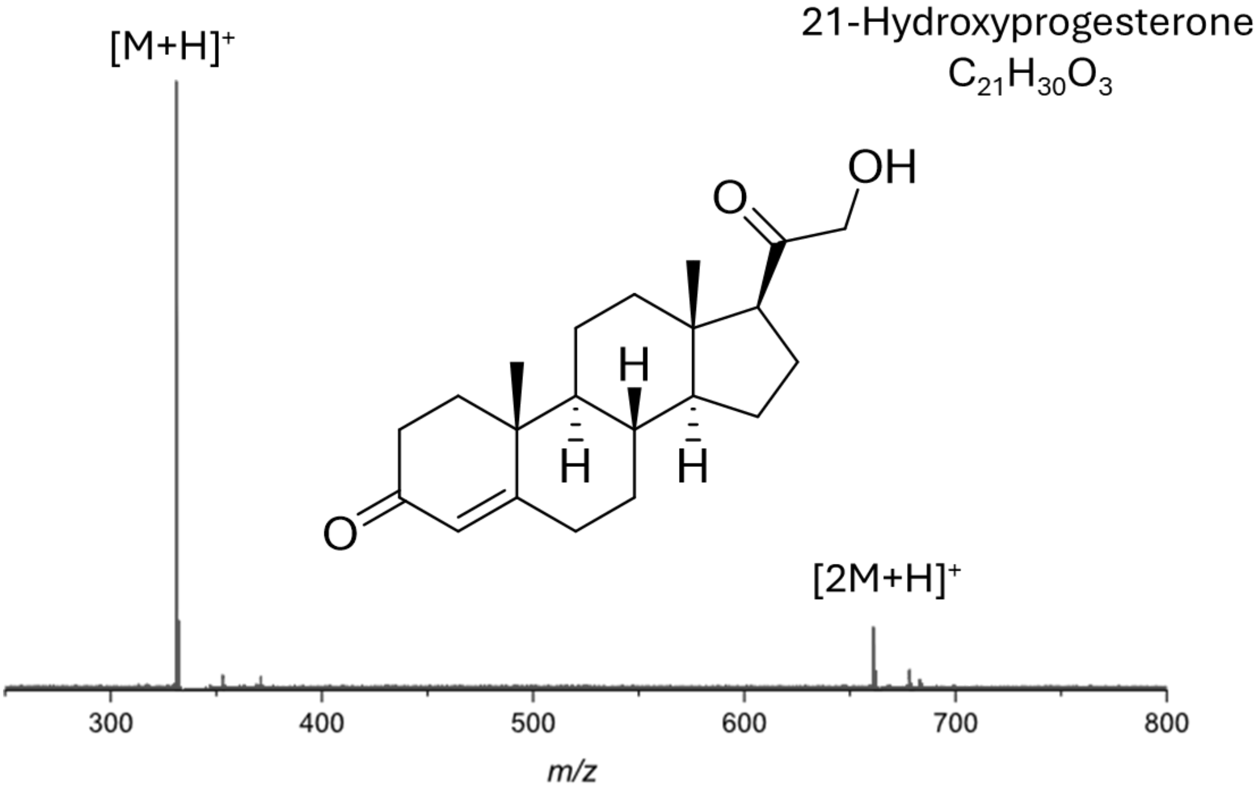

Figure S6: Mass spectrum of 21-hydroxyprogesterone in 1:1 methanol/water (v/v) with 0.1% formic acid (final concentration: 250 μM) when analysed on the cryogenic gas-phase IR-MS platform. The protonated dimer is also present.

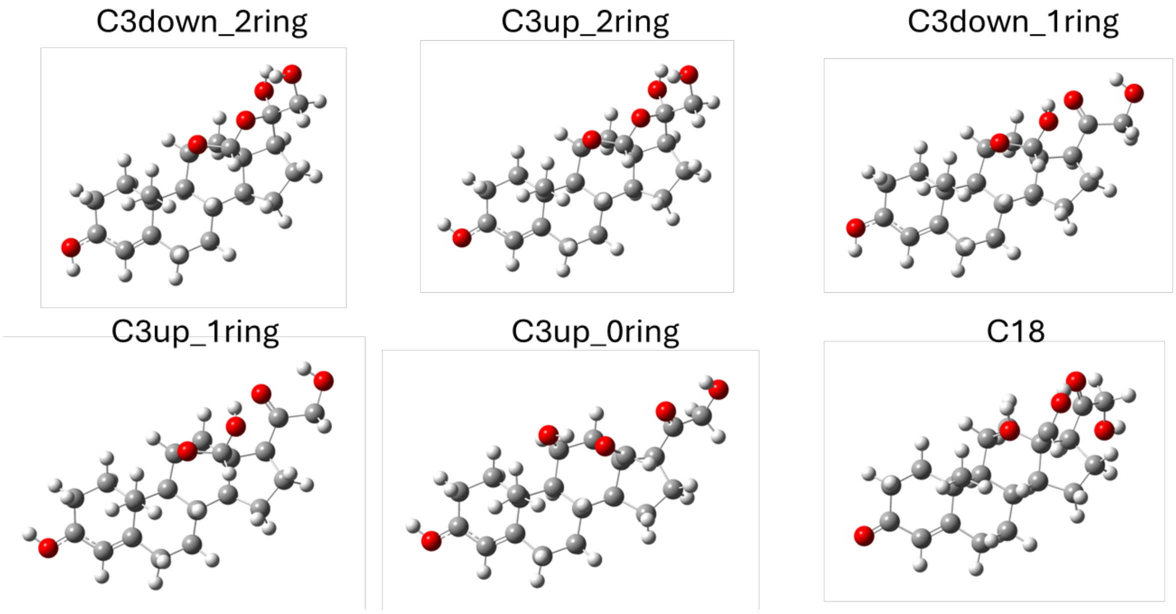

Figure S7: Lowest energy DFT optimised structures of aldosterone.

Table S2: Energetics of protonated aldosterone structures optimized at the PBE0+D3/6-311+G(d,p) level of theory. Energies (ΔE, including zero-point-vibrational energy) and free energies (ΔF) at 90 K are assigned to each structure. The infrared spectra of the structures labelled with an asterisk are represented in the manuscript. Structures that were identical after optimisation have been noted as a single structure to avoid duplicates.

ID	ΔE(PBE0 + D3) [kJ mol^{-1}]	ΔF(PBE0 + D3) [kJ mol^{-1}]
C3down_2ring (ald_protC3_taut1*)	0	0
C3up_2ring (ald_protC3_taut2*)	0.6800	0.6943
C3down_1ring (ald_protC3_taut3*)	11.3684	9.6423
C3up_1ring (ald_protC3_taut4*)	12.0248	10.3082
C3up_0ring (ald_protC3_1*)	32.4643	29.2577
ald_protC3_2	32.6796	29.3708
ald_protC3_3	32.6822	29.3718
ald_protC3_4	32.6822	29.3742
ald_protC3_5	32.6770	29.4513
ald_protC3_6	32.8161	29.4954
ald_protC3_7	32.8161	29.4962
ald_protC3_8	32.8161	29.4978
ald_protC3_taut5	32.4144	31.0180
ald_protC3_taut6	35.0557	33.9342
ald_protC3_9	36.9513	34.0451
ald_protC3_10	36.9539	34.0485
ald_protC3_11	36.9565	34.0519
ald_protC3_12	37.2322	34.2919
ald_protC3_13	37.2348	34.3048
ald_protC3_14	37.2375	34.3075
ald_protC3_15	37.2821	34.3703
ald_protC3_16	37.5709	34.6488
ald_protC3_17	42.9847	38.3388
ald_protC3_18	42.9847	38.3476
ald_protC3_19	42.9611	38.4975
ald_protC3_20	42.9611	38.4983
ald_protC3_21	43.4468	39.5198
ald_protC3_22	45.6391	42.4713
ald_protC3_23	45.6417	42.4763
ald_protC3_24	45.6443	42.4797
ald_protC3_25	45.6758	42.5667
ald_protC3_taut7	43.9640	42.7514
ald_protC3_26	47.0201	42.8957
ald_protC3_27	47.5531	43.2749
ald_protC3_28	47.6922	43.3919
C18 (ald_protC18_1*)	51.6016	49.7653

ald_protC3_29	54.5894	51.4272
ald_protC18_2	57.6822	55.3300
ald_protC18_3	59.3573	57.7588
ald_protC18_4	61.2634	58.9762
ald_protC18_5	61.5233	59.3486
ald_protC18_6	61.5260	59.3536
ald_protC3_taut8	65.1885	66.7784
ald_protC18_7	69.1058	67.0943
ald_protC18_8	69.3998	67.4851
ald_protC18_9	72.5268	70.9195
ald_protC18_10	74.2098	73.6716
ald_protC18_11	77.4417	75.1640
ald_protC18_12	78.4368	77.1030
ald_protC18_13	78.8779	78.2787
ald_protC18_14	78.8779	78.2795
ald_protC18_15	82.3068	81.9502
ald_protC18_16	86.8804	86.5602
ald_protC18_17	86.8830	86.5621
ald_protC3_taut8	101.3417	99.2264
ald_protC3_taut9	121.2850	121.1471
ald_protC3_taut10	125.7956	125.8471
ald_protC3_taut11	126.8274	126.3955
ald_protC3_taut12	128.0430	128.7143
ald_protC3_taut13	129.4923	129.4344
ald_protC3_taut14	129.9255	129.9184
ald_protC3_taut15	139.3615	139.5858
ald_protC3_taut16	141.7297	139.8110
ald_protC3_taut17	141.7297	139.8134
ald_protC3_taut18	142.2260	140.4000

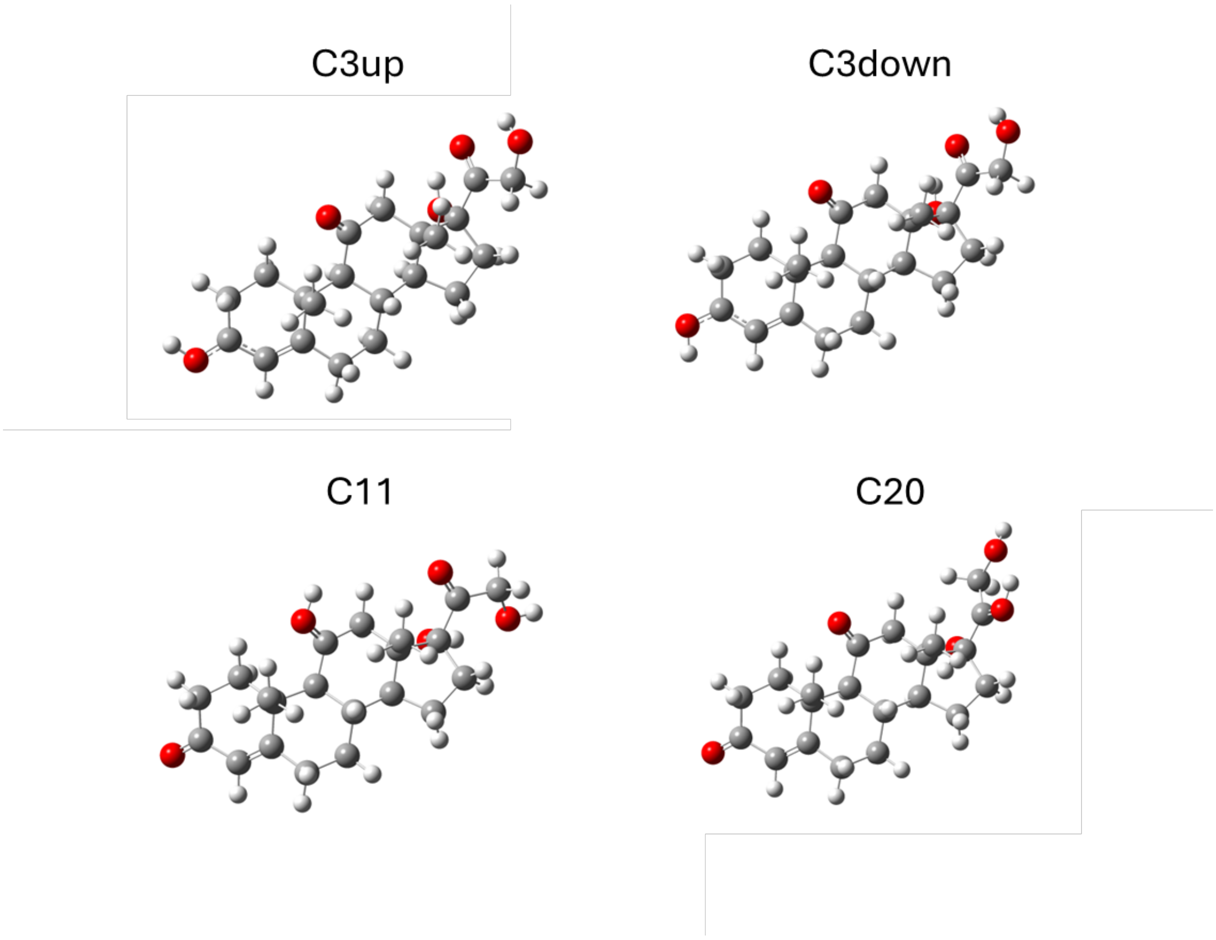

Figure S8: Lowest energy DFT optimised structures of cortisone.

Table S3: Energetics of protonated cortisone structures optimized at the PBE0+D3/6-311+G(d,p) level of theory. Energies (ΔE, including zero-point-vibrational energy) and free energies (ΔF) at 90 K are assigned to each structure. The infrared spectra of the structures labelled with an asterisk are represented in the manuscript.

ID	ΔE(PBE0 + D3) [kJ mol^{-1}]	ΔF(PBE0 + D3) [kJ mol^{-1}]
C3up (cort_protC3_1*)	0	0
C3down (cort_protC3_2*)	0.3334	0.3319
cort_protC3_3	2.8093	2.2799
cort_protC3_4	3.2346	2.7020
cort_protC3_5	3.2372	2.7039
cort_protC3_6	3.2372	2.7047
cort_protC3_7	5.4820	5.3624
cort_protC3_8	5.4873	5.3740
cort_protC3_9	6.2513	5.8233
cort_protC3_10	6.2539	5.8260
cort_protC3_11	6.6268	6.1940
cort_protC3_12	6.6320	6.1969
cort_protC3_13	6.3458	6.4711
cort_protC3_14	9.6986	9.3095
cort_protC3_15	9.9218	9.5350
cort_protC3_16	10.8564	10.5989
cort_protC3_17	11.0744	10.8200
cort_protC3_18	11.4708	11.0658
cort_protC3_19	11.4971	11.1016
cort_protC3_20	11.9066	11.3305
cort_protC3_21	11.4288	11.4296
cort_protC3_22	12.0931	11.5248
cort_protC3_23	11.9171	11.6849
cort_protC3_24	11.7465	11.7243
cort_protC3_25	12.2296	12.1226
cort_protC3_26	12.2322	12.1244
cort_protC3_27	12.6890	12.5702
cort_protC3_28	16.9161	16.8186
cort_protC3_29	17.2180	16.8630
cort_protC3_30	16.9502	16.8694
cort_protC3_31	17.3703	16.9225
cort_protC3_32	19.6282	19.7749
cort_protC3_taut1	27.5179	27.2001
cort_protC3_taut2	27.9406	27.9945
cort_protC3_taut3	61.7386	61.5278
C11 (cort_protC11_1*)	63.0409	62.5352
cort_protC11_2	63.0540	62.7941
cort_protC11_3	67.9217	67.9946

cort_protC11_4	68.9798	68.8688
C20 (cort_protC20_1*)	70.1455	69.3038
cort_protC20_2	70.1507	69.3106
cort_protC11_5	69.4103	69.4476
cort_protC11_6	70.2216	70.0195
cort_protC11_7	73.2830	72.6299
cort_protC11_8	74.3227	73.8249
cort_protC11_9	75.4884	74.5984
cort_protC11_10	74.5695	74.9419
cort_protC11_11	76.2892	75.8580
cort_protC20_3	76.5491	75.9158
cort_protC11_12	76.2813	76.1275
cort_protC11_13	77.4916	76.5002
cort_protC11_14	78.5445	77.1242
cort_protC11_15	78.9724	77.8335
cort_protC11_16	78.9750	77.8362
cort_protC11_17	79.4187	77.9533
cort_protC11_18	78.7597	78.1503
cort_protC11_19	79.1588	78.2323
cort_protC11_20	79.2796	78.6836
cort_protC11_21	80.3954	79.7884
cort_protC11_22	79.9806	80.1637
cort_protC11_23	81.1411	81.2275
cort_protC11_24	82.4827	82.4383
cort_protC11_25	84.1131	83.8136
cort_protC11_26	84.8378	84.0413
cort_protC11_27	84.7459	84.0913
cort_protC11_28	84.6172	84.3113
cort_protC11_29	85.3970	85.0348
cort_protC11_30	85.9300	85.2952
cort_protC20_4	88.0383	87.7236
cort_protC20_5	90.7557	90.4006
cort_protC20_6	96.0565	95.2212
cort_protC3_33	122.9837	122.3465

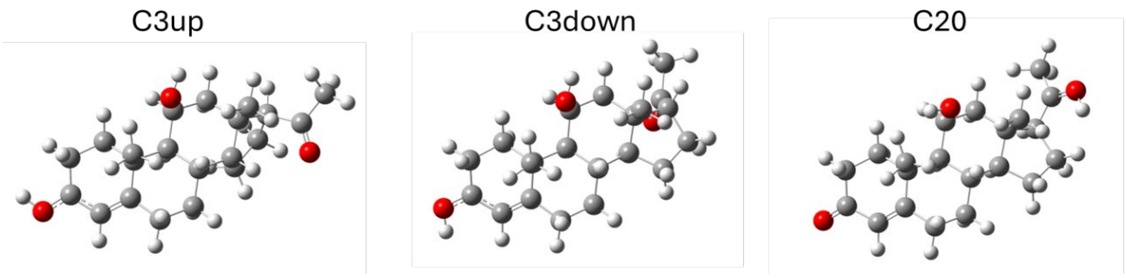

Figure S9: Lowest energy DFT optimised structures of 11β-hydroxyprogesterone.

Table S4: Energetics of protonated 11β-hydroxyprogesterone structures optimized at the PBE0+D3/6-311+G(d,p) level of theory. Energies (ΔE, including zero-point-vibrational energy) and free energies (ΔF) at 90 K are assigned to each structure. The infrared spectra of the structures labelled with an asterisk are represented in the manuscript.

ID	ΔE(PBE0 + D3) [kJ mol^{-1}]	ΔF(PBE0 + D3) [kJ mol^{-1}]
C3up (11b_protC3_1*)	0	0
C3down (11b_protC3_2*)	0.4385	0.4353
11b_protC3_3	1.6698	1.6770
11b_protC3_4	1.7853	1.8170
11b_protC3_5	2.3971	2.4478
11b_protC3_6	5.2510	5.6425
11b_protC3_7	5.2536	5.6451
11b_protC3_8	6.2093	6.6064
11b_protC3_9	6.2093	6.6072
11b_protC3_10	7.1939	6.9965
11b_protC3_11	7.1939	6.9973
11b_protC3_12	8.0970	7.9092
11b_protC3_13	10.7646	10.4317
11b_protC3_14	11.3763	11.1108
11b_protC3_15	12.5053	12.6384
11b_protC3_16	14.7894	14.8211
11b_protC3_17	14.9102	15.0648
11b_protC3_18	15.6138	15.7613
11b_protC3_19	15.8528	15.9185
11b_protC3_20	19.9669	19.9756
11b_protC3_21	19.9696	19.9767
11b_protC3_22	20.9961	21.0001
11b_protC3_23	20.9987	21.0035
11b_protC3_24	42.5095	42.6141
11b_protC3_taut_1	49.1704	49.2258
C20 (11b_protC20_1*)	62.9227	62.8570
11b_protC20_2	62.9227	62.8577
11b_protC20_3	62.9254	62.8596
11b_protC20_4	65.9263	65.6624
11b_protC20_5	65.9263	65.6640
11b_protC20_6	66.2676	66.4650
11b_protC20_7	66.5407	66.6833
11b_protC20_8	66.5407	66.6841
11b_protC3_taut_2	67.1970	67.9056
11b_protC20_9	68.0372	68.1751
11b_protC20_10	68.0451	68.1909
11b_protC20_11	69.6834	69.6913
11b_protC20_12	69.9827	69.9621

11b_protC20_13	69.9827	69.9629
11b_protC20_14	71.0670	71.0480
11b_protC20_15	71.3742	71.7911
11b_protC20_16	73.5245	73.1908
11b_protC20_17	73.1517	73.3451
11b_protC20_18	73.2357	73.4449
11b_protC20_19	74.9160	75.2299
11b_protC20_20	76.0450	76.1028
11b_protC20_21	76.4624	77.0061
11b_protC20_22	77.1897	77.0772
11b_protC20_23	79.8441	80.2126
11b_protC20_24	81.2750	80.8288
11b_protC20_25	83.3045	83.1595
11b_protC20_26	88.6763	88.3743
11b_protC20_27	91.0655	90.8539
11b_protC20_28	91.5118	91.5110
11b_protC20_29	93.4914	93.6943
11b_protC3_25	108.9740	106.9300
11b_protC3_26	109.0606	107.7744
11b_protC3_27	131.8264	130.9221
11b_protC3_28	131.5270	131.4208
11b_protC3_29	131.7975	131.6112
11b_protC3_30	137.8519	137.4295
11b_protC3_31	138.0462	137.5936
11b_protC20_30	155.2012	154.6306
11b_protC20_31	168.5518	167.9139
11b_protC20_32	168.5545	167.9165
11b_protC20_33	173.6348	173.3313

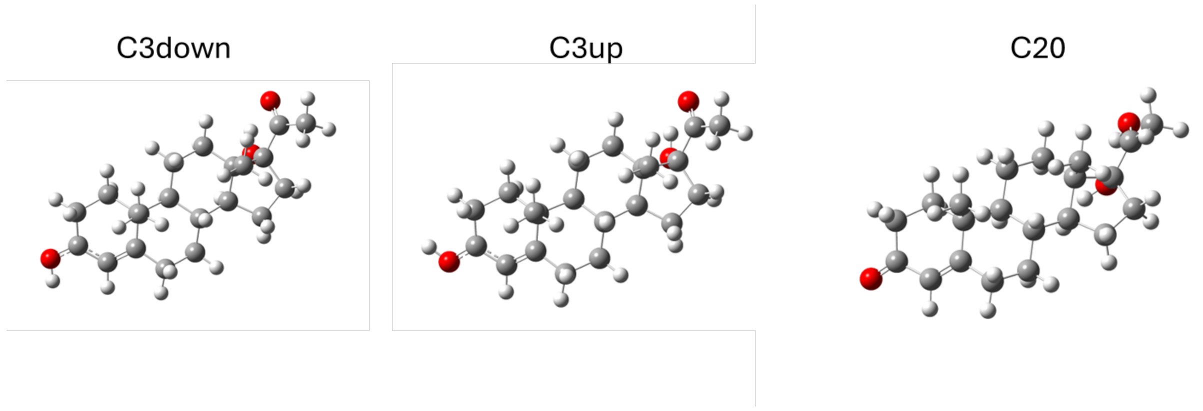

Figure S10: Lowest energy DFT optimised structures of 17α-hydroxyprogesterone.

Table S5: Energetics of protonated 17α-hydroxyprogesterone structures optimized at the PBE0+D3/6-311+G(d,p) level of theory. Energies (ΔE, including zero-point-vibrational energy) and free energies (ΔF) at 90 K are assigned to each structure. The infrared spectra of the structures labelled with an asterisk are represented in the manuscript.

ID	ΔE(PBE0 + D3) [kJ mol^{-1}]	ΔF(PBE0 + D3) [kJ mol^{-1}]
C3down (17a_protC3_1*)	0	0
17a_protC3_2	0.0026	0.0034
17a_protC3_3	0.0026	0.0042
C3up (17a_protC3_4*)	1.0712	1.0696
17a_protC3_5	1.8930	2.3598
17a_protC3_6	2.9327	3.4169
17a_protC3_7	4.8624	4.4899
17a_protC3_8	5.9546	5.2540
17a_protC3_9	5.9573	5.2559
17a_protC3_10	7.0311	6.3305
17a_protC3_11	8.6143	8.4375
17a_protC3_12	13.2929	12.7857
17a_protC3_13	14.6057	14.0834
17a_protC3_14	15.5036	14.2117
17a_protC3_15	15.5036	14.2197
17a_protC3_16	16.3700	15.0718
17a_protC3_17	16.3700	15.0782
17a_protC3_18	16.5249	16.5249
17a_protC3_19	17.5436	16.6409
17a_protC3_20	17.6591	16.7493
17a_protC3_21	18.2945	17.3759
17a_protC3_22	18.2945	17.3799
17a_protC3_23	18.3759	17.4058
17a_protC3_24	18.0766	18.0575
17a_protC3_25	18.8721	19.0916
17a_protC3_26	18.8747	19.0958
17a_protC3_27	20.1297	20.3469
17a_protC3_28	25.4647	24.9987
17a_protC3_29	26.6987	26.2264
C20 (17a_protC20_1*)	49.1756	49.2176
17a_protC20_2	49.1756	49.2184
17a_protC3_taut_1	54.1089	54.6138
17a_protC20_3	55.9153	56.1253
17a_protC20_4	55.9205	56.1369
17a_protC20_5	55.9205	56.1393
17a_protC20_6	73.8711	73.6848
17a_protC20_7	73.8711	73.6872
17a_protC20_8	73.8789	73.7006

17a_protC20_9	74.6167	74.7332
17a_protC20_10	74.6167	74.7340
17a_protC20_11	76.1264	75.5486
17a_protC20_12	76.1290	75.5520
17a_protC20_13	76.8248	76.6242
17a_protC20_14	76.8274	76.6285
17a_protC20_15	76.8300	76.6350
17a_protC20_16	80.7683	80.8261
17a_protC20_17	82.8161	82.3375
17a_protC20_18	83.7009	83.7873
17a_protC3_30	127.8382	127.8160
17a_protC3_31	127.8408	127.8210
17a_protC20_19	157.2727	156.5499
17a_protC20_20	165.1413	164.2109

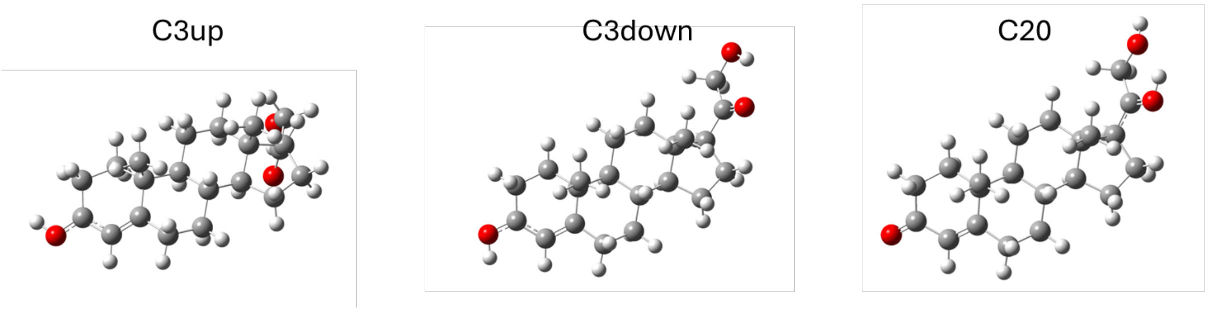

Figure S11: Lowest energy DFT optimised structures of 21-hydroxyprogesterone.

Table S6: Energetics of protonated 21-hydroxyprogesterone structures optimized at the PBE0+D3/6-311+G(d,p) level of theory. Energies (ΔE, including zero-point-vibrational energy) and free energies (ΔF) at 90 K are assigned to each structure. The infrared spectra of the structures labelled with an asterisk are represented in the manuscript.

ID	ΔE(PBE0 + D3) [kJ mol^{-1}]	ΔF(PBE0 + D3) [kJ mol^{-1}]
C3up (21_protC3_1*)	0	0
C3down (21_protC3_2*)	5.2142	5.0074
21_protC3_3	6.2461	6.0448
21_protC3_4	7.0442	6.8627
21_protC3_5	7.9054	7.7255
21_protC3_6	7.9605	7.9201
21_protC3_7	9.1079	9.3139
21_protC3_8	10.2473	10.4621
21_protC3_9	10.2920	10.5004
21_protC3_10	10.2893	10.5057
21_protC3_11	11.4288	10.5689
21_protC3_12	11.2188	11.4415
21_protC3_13	13.2036	12.3810
21_protC3_14	13.2141	12.3931
21_protC3_15	14.4140	13.5802
21_protC3_16	14.7264	14.1138
21_protC3_17	15.3907	14.7765
21_protC3_18	15.8423	15.2431
21_protC3_19	16.0156	15.6645
21_protC3_20	16.0182	15.6687
21_protC3_21	16.3752	15.7658
21_protC3_22	17.3125	16.9250
21_protC3_23	17.6381	17.4463
21_protC3_24	18.0582	17.9100
21_protC3_25	18.8275	18.2093
21_protC3_26	18.8170	18.6323
21_protC3_27	19.1399	19.0067
21_protC3_28	19.8462	19.2296
21_protC3_29	21.2272	19.7435
21_protC3_30	22.5819	21.2544
21_protC3_31	21.6210	21.3571
21_protC3_32	22.8576	22.6278
21_protC3_33	25.8454	25.0458
21_protC3_34	28.2320	27.5869
21_protC3_35	28.6180	28.1757
21_protC3_taut_1	36.1846	36.0769
21_protC3_taut_2	40.9972	40.5962
C20(21_protC20_1*)	49.7191	49.8514
21_protC3_36	52.5074	53.4156
21_protC20_2	53.4709	53.9045
21_protC20_3	53.4709	53.9052

21_protC3_taut_3	56.4535	55.6388
21_protC20_4	56.6767	57.0809
21_protC20_5	56.6819	57.0885
21_protC3_taut_4	58.3150	57.8307
21_protC20_6	57.9212	57.8950
21_protC20_7	60.1450	60.7782
21_protC20_8	60.1476	60.7808
21_protC20_9	66.5380	66.9478
21_protC20_10	74.3804	74.3495
21_protC20_11	75.2573	75.4253
21_protC20_12	75.2573	75.4261
21_protC20_13	78.6994	79.1725
21_protC20_14	80.9940	81.1121
21_protC20_15	80.9967	81.1171
21_protC20_16	81.3065	81.8557
21_protC20_17	85.8722	85.7993
21_protC3_37	125.7352	125.7701
21_protC3_38	129.0223	129.0366
21_protC3_39	136.8699	136.7740
21_protC3_40	142.3914	141.4261
21_protC20_18	156.5349	155.8763
21_protC20_19	172.2591	171.4055
